# Multiple anti-epileptic drug use in children with epilepsy in Mulago hospital, Uganda: a cross sectional study

**DOI:** 10.1186/s12887-016-0575-0

**Published:** 2016-03-09

**Authors:** Rita Atugonza, Angelina Kakooza-Mwesige, Samden Lhatoo, Mark Kaddumukasa, Levicatus Mugenyi, Martha Sajatovic, Elly Katabira, Richard Idro

**Affiliations:** Department of Pediatrics, Makerere University, College of Health Sciences, Kampala, 7072 Uganda; Neurological and Behavioral Outcomes Center, University Hospital Case Medical Center, Case Western Reserve University, 11100 Euclid Ave, Cleveland, OH 44106 USA; Department of Medicine, Makerere University, College of Health Sciences, Kampala, 7072 Uganda; Infectious Diseases Research Collaboration, Mulago Hospital Complex, Kampala, Uganda; Centre for Statistics, Interuniversity Institute for Biostatistics and Statistical Bioinformatics, Hasselt University, Diepenbeek, Belgium; Centre for Tropical Medicine and Global Health, Nuffield Department of Medicine, University of Oxford, Oxford, UK

**Keywords:** Epilepsy, Therapy, Anti-epileptic drugs, Children

## Abstract

**Background:**

Seizures in up to one third of children with epilepsy may not be controlled by the first anti-epileptic drug (AED). In this study, we describe multiple AED usage in children attending a referral clinic in Uganda, the factors associated with multiple AED use and seizure control in affected patients.

**Methods:**

One hundred thirty nine patients attending Mulago hospital paediatric neurology clinic with epilepsy and who had been on AEDs for ≥6 months were consecutively enrolled from July to December 2013 to reach the calculated sample size. With consent, the history and physical examination were repeated and the neurophysiologic and imaging features obtained from records. Venous blood was also drawn to determine AED drug levels. We determined the proportion of children on multiple AEDs and performed regression analyses to determine factors independently associated with multiple AED use.

**Results:**

Forty five out of 139 (32.4 %) children; 46.7 % female, median age 6 (IQR = 3–9) years were on multiple AEDs. The most common combination was sodium valproate and carbamazepine. We found that 59.7 % of children had sub-therapeutic drug levels including 42.2 % of those on multi-therapy. Sub-optimal seizure control (adjusted odds ratio [OR^a^] 3.93, 95 % CI 1.66–9.31, *p* = 0.002) and presence of focal neurological deficits (OR^a^ 3.86, 95 % CI 1.31–11.48, *p* = 0.014) were independently associated with multiple AED use but not age of seizure onset, duration of epilepsy symptoms, seizure type or history of status epilepticus.

**Conclusion:**

One third of children with epilepsy in Mulago receive multiple AEDs. Multiple AED use is most frequent in symptomatic focal epilepsies but doses are frequently sub-optimal. There is urgent need to improve clinical monitoring in our patients.

## Background

Epilepsy contributes 10 % of the global burden of brain disorders [1], and is associated with considerable morbidity and mortality [2] and poor quality of life. Worldwide, up to 80 million people are affected of whom 10.5 million are children <15 years [3]. In Uganda, the estimated prevalence of epilepsy is 10 · 3 (9 · 5-11 · 1) per 1000 population [4] and the age specific prevalence rate in children <15 years is 2.0 % [5].

Despite its debilitating effects, over 70 % of patients can attain good seizure control with appropriate treatment. The goal of treatment is restoration of near normal life with complete seizure control using a single anti-epileptic drug (AED). Monotherapy is recommended because of fewer adverse drug effects, absence of drug-drug interactions, better compliance, and lower cost compared to therapy with multiple AEDs [6–9]. Studies in developed countries with adequate resources for treatment have however shown that 17-40 % of children do not respond to the first drug used and may require multiple AEDs [10, 11]. It has been suggested that the patients’ clinical characteristics such as frequent, focal and long duration of seizures, symptomatic or syndromic epilepsy, history of status epilepticus, and the presence of neurological deficits, is the primary reason for failure of the first AED, rather than drug related factors such as efficacy and adverse effects. The answers to these questions are important, because inadequate response to initial treatment with the first AED and subsequent treatment with multiple AEDs is believed, in itself, to be a poor prognostic factor in epilepsy [12–14].

The main objective of the study was to determine the use of multiple AEDs and associated factors among children attending a referral clinic in Uganda.

## Methods

### Study design

This was a cross sectional descriptive study of one hundred thirty nine children with epilepsy attending Mulago hospital in Kampala, Uganda.

### Setting

The study was carried out in the paediatric neurology clinic (PNC) at Mulago hospital in Kampala, Uganda. Mulago hospital is a public hospital located 2 km from the city center and serves as a National Referral for the entire country and a general hospital as well as Health Center IV, III for the Kampala metropolitan area (Uganda’s capital city) with an official bed capacity of 1790. It also serves as a teaching hospital for Makerere University College of Health Sciences. The PNC is under the Department of Paediatrics and Child Health and is run as an outpatient specialized clinic which caters to children with neurological disorders once a week every Thursday between 8 am – 3 pm. It serves as a referral outpatient clinic for the neurological cases from all over the country. Annually the clinic sees about 300 new patients and on each clinic day 25 – 40 children with ages ranging from 2 months to 18 years are attended to; the clinic´s upper age limit is 16 but there are older patients who have not yet been transferred to the adult clinic. Epilepsy is the most common diagnosis in 68.4 % of the children attending the PNC. Patients are seen by a team comprised of a paediatric neurologist, paediatricians, residents, nurses and a records clerk. Children seen in this clinic are referrals from paediatric wards and other hospitals around the country with a few self-referrals. Services provided include; clinical evaluation and care, referrals to specialized clinics, laboratory tests- HIV rapid test, blood slide for malaria parasites and haemoglobin estimation. Other investigations such as renal function and liver function tests, limited biochemical tests and other specific tests are done in the hospital’s main laboratory. Serum drug level tests are not done at the hospital but may be sourced at a fee from privately run laboratories. Investigations such as Electro encephalogram (EEG) and CT scans are also carried out in the hospital at a cost. Magnetic resonance imaging (MRI) is available outside the hospital for patients who can afford it.

In Uganda, treatment is offered free of charge in all health centers. Both a national treatment guideline and a national list of essential medicines exist to aid the health personnel in the management of epilepsy but these are not readily available even at this clinic. Most epilepsy patients receive the older generation AEDs which are given after consultation with a paediatric neurologist but the choice of drug eventually given is usually based on availability. These AEDs might be initiated as part of the hospitalization or at the provider’s discretion before referrals to the PNC are made and may require adjustments during the clinic visits. Subsequent increment of doses and use of additional AEDs is usually at the prescriber’s discretion. Patients requiring some of the new generation AEDs have to source for these privately at a cost. Patients are given appointments of 2 weeks up to 3 months based on seizure control and also to monitor the side effects of the AEDs.

### Participants

The study included participants with a diagnosis of epilepsy (two or more unprovoked seizures); aged <18 years; had received AEDs for at least six months before enrolment into the study and whose parents or guardians provided written informed consent. In addition, children older than eight years and without severe mental retardation provided assent. Acutely ill children requiring hospitalization and children being treated with any of newer generation AEDs (lamotrigine, levetiracetam, vigabatrin and topiramate) were excluded due to limitations in measuring the serum drug levels in the country.

### Sample size

The sample size was estimated using the Daniel 1999 formula for finite populations and was based on a prevalence multiple drug use of 20 % by Carpay et al. [15] A sample size of 136 patients was attained.

### Study procedures

The study was approved by Makerere University School of Medicine Research and Ethics Committee, reference 2013–063.

- Recruitment
  On each clinic day from July to December 2013, patients with epilepsy and fulfilling the study eligibility criteria were approached for possible participation and those with consenting parents/guardians were consecutively enrolled. Unattended minors were given consent forms to take home and asked to return them on scheduled appointment dates when they were interviewed with their parents/guardians.
- Data collection
  For the history, information from patient’s records was abstracted on to a case record form. This was supplemented by direct inquiry. Data thus collected included the socio-demographic characteristics, specific clinical details such as age of onset of seizures, seizure types, frequency and duration, epilepsy diagnosis,, possible risk factors in the birth and past medical history, and AED history (type of drugs, doses, adverse events, duration of treatment, treatment response, and adherence). In addition, reports of electroencephalographic (EEG) recordings and brain imaging (computerized tomography [CT] and magnetic resonance imaging [MRI] scans) and the conclusions from these reports e.g. the clinical classification of the epilepsy were abstracted when available. Seizures were broadly categorized according to the 2010 International League against Epilepsy (ILAE) criteria into generalized, focal and unclassified type seizures [15].
  A physical examination was performed to assess the nutritional status, identify neurological deficits; describe function and concurrent co-morbidities. The height and weight was measured, patients had an examination for peripheral stigmata of central nervous system disease and a comprehensive examination of all systems. The neurologic examination documented the mental status, assessed the cranial nerves and motor deficits and classified this using a topographical classification system – (monoparesis, diplegia, hemiparesis or quadriparesis). Abnormalities of movement and coordination such as tremors, chorea, athetosis, dystonia, gait and ataxia were also recorded.
- Determination of drug levels
  Two mls of venous blood was drawn from a peripheral vein to determine the drug levels at study enrolment. The collected sample was placed into plain vacutainer tubes between 9.00 am and 1.00 pm on the day of enrolment. The samples were then transported on ice to the Lancet laboratories, an internationally accredited laboratory by the South African National Accreditation System (SANAS) for analysis within 8 hours of collection. In the laboratory, the samples were centrifuged at 3000 rpm for 10 minutes and sera collected. The serum levels of all the older generation AEDs (carbamazepine, phenobarbitone and sodium valproate) were then determined using a Fluorescence Polarization Immunoassay (FPIA) method using COBAS® 4000 analyzer, Roche Diagnostics. The FPIA method offers significant advantages in calibration curve stability while maintaining accuracy and precision comparable with those of established HPLC procedures. The individual therapeutic range of carbamazepine was 34–51 μmol/l when used as monotherapy and 17–34 μmol/l if it was part of a combination therapy. For phenobarbitone, the therapeutic range was 43–172 μmol/l while for sodium valproate this was 347–693 μmol/l irrespective of whether each was being used alone or as part of combination therapy [16–18].

### Data management and analysis

The case record forms were cross- checked for completeness before end of the day and the data entered into an Epidata version 3.1(Odense, Denmark) database. Data was analyzed using STATA version 12.0, (STATA Corporation, TX). The proportion of children being treated with multiple AED was determined. In examining factors associated with the use of multiple AED as opposed to monotherapy, categorical variables were summarized as proportions/percentages and compared using the chi-square test or fisher’s exact test. Normally distributed continuous data were summarized using the mean with standard deviations and compared using the student’s t test while the median was used for skewed data. Variables with *p*-values ≤ 0.2 at bivariate analysis were subjected to logistic regression analysis to identify factors independently associated with multiple AED therapy. A *p*-value ≤ 0.05 was considered statistically significant. To examine the relationship between multiple AED use and seizure control we used the chi square test for trends to analyze the differences in the frequency of seizures in children on monotherapy compared to those on multiple AED therapy. Good seizure control was defined as ≤ 1 seizure in the previous 6 months.

## Results

### General descriptions

A total of 215 children aged less than 18 years attending the neurology clinic during the study period were screened. The majority (163/215, 75.8 %) had epilepsy. Twenty four epilepsy patients were excluded; 23 had been on treatment for less than six months and 1 was being treated with lamotrigine. We subsequently enrolled 139 children into this study. The median age of the participants was 6 (IQR 4–10) years; 78/139 (56 %) were male; the median age of onset of seizures was 1 (IQR 0.3 – 4) years and the median duration of seizures was 4 (IQR 2 – 5) years (Table 1).

**Table 1:** Demographics of study participants

Characteristics	Multi therapy	Mono therapy	Odds Ratio
	*N* = 45 (%)	*N* = 94 (%)	(95 % CI)
Male	24 (53.4)	54 (57.5)	1
Female	21 (46.6)	40 (42.5)	1.18 (0.58 – 2.41)
0 – 1 years	2 (4.5)	6 (6.4)	1
2 – 6 years	23 (51.1)	42 (44.7)	1.64 (0.31 – 8.81)
6 – 13 years	18 (40.0)	38 (40.4)	1.42 (0.26 – 7.75)
14 – 18 years	2 (4.4)	8 (8.5)	0.75 (0.08 – 6.96)
Primary caretaker			
Mother	30 (66.6)	66 (70.2)	1
Father	6 (13.3)	11 (11.7)	1.20 (0.41 – 3.55)
Grandparent	1 (2.2)	8 (8.5)	0.28 (0.03 – 2.30)
Uncle/Aunt	5 (11.1)	6 (6.4)	1.83 (0.52 – 6.48)
Other	3 (6.8)	3 (3.2)	2.20 (0.42 – 11.54)
District			
Kampala	11 (24.4)	28 (29.8)	1
Wakiso	22 (48.9)	46 (48.9)	1.22 (0.51 – 2.88)
Others	12 (26.7)	20 (21.3)	1.53 (0.56 – 4.15)
Guardian Education Level			
No formal education	4 (8.9)	3 (3.3)	1
Primary level	19 (42.2)	38 (41.3)	0.38 (0.08 – 1.85)
Secondary level	13 (28.9)	43 (46.7)	0.23 (0.04 – 1.15)
Tertiary institution	9 (20.0)	8 (8.7)	0.84 (0.14 – 4.97)
Mother employment			
Unemployed	25 (55.6)	36 (39.2)	1
Casual laborer	3 (6.7)	4 (4.4)	1.08 (0.22 – 5.25)
Self-employed	11 (24.4)	42 (45.6)	0.38 (0.16 – 0.87)
Formally employed	6 (13.3)	10 (10.8)	0.86 (0.28 – 2.68)
Household monthly income			
Less than100,000	9 (19.9)	27 (28.7)	1
100,000 - 200,000	12 (26.7)	26 (27.7)	1.38 (0.50 – 3.83)
200,000 - 500,000	12 (26.7)	16 (17.0)	2.25 (0.78 – 6.51)
Greater than 500,000	12 (26.7)	25 (26.6)	1.44 (0.52 – 4.00)

### Patterns of epilepsy

Based on EEGs, generalized epilepsy was described in 49/125 (39.2 %) children; 57.1 % were male with a median age of 6 (IQR = 4 – 8) years. Focal epilepsy in 59/125 (47.2 %) children; 50.8 % were male with a median age of 7 (IQR = 4 – 12) years while 17/125 (13.6 %) had epileptic syndromes; 58.8 % were male with a median age of 4 (IQR = 3 – 7) years. (13 - Lennox-Gastaut syndrome, 2 - Infantile spasms and 2 - Benign Rolandic epilepsy). Fourteen children did not have EEG tracings or reports. Overall epilepsy was symptomatic or probably symptomatic in 95/139 (68.4 %) participants, idiopathic in 11/139 (7.9 %) and cryptogenic in 33/139 (23.7 %). Patients were considered to have symptomatic or probably symptomatic epilepsy if they had a history of known or suspected risk factor for epilepsy. Idiopathic epilepsy was considered for those with epileptic syndromes without a history of known or suspected risk factor. These included; absence epilepsy, benign rolandic epilepsy with centrotemporal spikes and myoclonic epilepsy. Cryptogenic epilepsy was considered for those who did not meet the criteria for idiopathic or symptomatic categories [19].

### Neurological impairments among the study participants

**Fig. 1:**
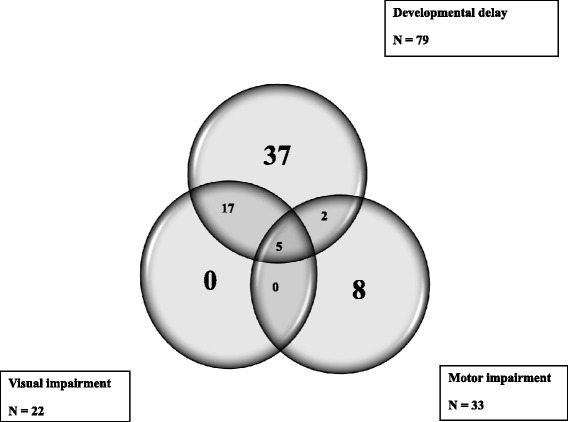
Distribution of neurological impairments among the study participants. Majority of the children had developmental delays (*n* = 79) compared to motor (*n* = 33) and visual impairment (*n* = 28)

The study participants were assessed for development delay, visual, motor and hearing impairments. Ninety four children (67.6 %) had a neurological deficit; 42 % female, median age 5.5 (IQR = 3 – 8) years. Seventy nine children 79/94 (84.0 %) had developmental delay in at least one field; gross motor, fine motor, speech/language and social skills. The frequency of participants with a single impairment was thirty three 33/94 (35.1 %), (Fig. 1).

### Use of multiple anti-epileptic drugs

Forty five of the 139 children (32.4 %) were on multiple AED; 42 were on dual therapy while three were on triple therapy. The most common drug combination was sodium valproate and carbamazepine, 34/45 (75.5 %) followed by carbamazepine and phenobarbitone, 6/45 (13.4 %) and sodium valproate and phenobarbitone, 2/45 (4.5 %). Of the 94/139 participants on monotherapy, 54/139 (38.8 %) were on carbamazepine only, 37/139 (26.6 %) were on sodium valproate only, two were on phenobarbitone and one was on phenytoin only. Regarding seizure control; thirteen children on multi AED therapy, 13/45 (28.9 %) had good seizure control compared to 61/94 (64.9 %) patients on monotherapy.

### Clinical characteristics and multiple anti-epileptic drug use

At bivariate analysis, patients with poor seizure control (defined as ≥ 1 seizure in past 6 months), the presence of a neurological deficit and a history of status epilepticus were more likely to be using multiple AED therapy (Table 2).

**Table 2:** Clinical characteristics of study participants

Variables		Outcome		Unadjusted Odds ratio (CI)	p-value
		Multi therapy *N* = 45 (%)	Mono therapy *N* = 94 (%)		
Age at onset	<1 year	22 (48.9)	35 (37.2)	1.61 (0.79 - 3.31)	0.193
	≥1 year	23 (51.1)	59 (62.8)	1	
Duration of epilepsy	<2 years	4 (8.9)	17 (18.1)	1	
	≥2 years	41 (91.1)	77 (81.9)	2.26 (0.71 – 7.17)	0.165
Seizure pattern^a^	Generalized	12 (29.3)	37 (41.7)	1	
	Focal	24 (58.5)	35(41.7)	2.11 (0.92 – 4.86)	0.078
	Epileptic syndrome	5 (12.2)	12 (14.3)	1.28 (0.38 – 4.39)	0.690
Seizure frequency^b^	≥1seizure/day	29 (64.5)	45 (47.9)	1	
	≥1 seizure/week	2 (4.5)	10 (10.6)	0.32 (0.06 – 1.55)	
	≥1 seizure/month	10 (22.2)	29 (30.9)	0.53 (0.23 – 1.24)	0.317
	≤1 seizure/year	4 (8.8)	10 (10.6)	0.63 (0.18 – 2.21)	
Neurological deficit	No	5 (11.1)	40 (42.5)	1	
	Yes	**40 (88.9)**	**54 (57.5)**	**5.93 (2.15 – 16.36)**	**<0.001**
Symptomatic epilepsy	No	11 (24.4)	33 (35.1)	1	
	Yes	34 (75.6)	61 (64.9)	1.67 (0.75 – 3.73)	0.240
Prior history of status epilepticus	No	28 (62.2)	75 (79.8)	1	
	Yes	**17 (37.8)**	**19 (20.2)**	**2.40 (1.09 – 5.26)**	**0.027**
Family history of epilepsy	No	30 (66.7)	75 (79.8)	1	
	Yes	15 (33.3)	19 (20.2)	1.97 (0.89 – 4.39)	0.092
Seizure control	Good	13 (28.9)	61 (64.9)	1	
	Poor	**32 (71.1)**	**33 (35.1)**	**4.55 (2.10 – 9.84)**	**<0.001**

^a^125 children had EEG records ^b^Seizure frequency before initiation of treatment

### Treatment history and multiple anti-epileptic drug use

Children initially treated with two drugs (17/139, *p* = 0.016) were more likely to be using multiple AEDs while children using sodium valproate alone were less likely to be on multiple drugs, (Table 3).

**Table 3:** Treatment history of study participants

Variables		Outcome		Unadjusted OR (95 % CI)	P-value*
		Multi therapy 45 (%)	Mono therapy 94 (%)		
Time to seek treatment	<1 week	3 (6.7)	10 (10.6)	1	
	1 Week < 1 month	6 (13.3)	6 (6.4)	3.33 (0.59 – 18.5)	0.336
	1 Month < 1 year	13 (28.9)	37 (39.4)	1.14 (0.27 – 4.79)	
	≥1 year	23 (51.1)	41 (43.6)	1.82 (0.46 – 7.31)	
First treatment point	Government health center	4 (8.9)	3 (3.2)	1	
	General hospital	4 (8.9)	1 (1.1)	4 (0.29 – 53.5)	0.065
	National referral hospital	33 (73.3)	79 (84.0)	0.41 (0.10 – 1.75)	
	Private clinic	4 (8.9)	6 (6.4)	0.67 (0.10 – 4.35)	
	Traditional healer	0 (0.0)	5 (5.3)	-	
Initial no. of drugs given to the child	One	35 (77.8)	87 (92.6)	1	
	Two	10 (22.2)	7 (7.4)	3.55 (1.25 – 10.07)	**0.013**
Child using Valproate	Yes	9 (20.0)	37 (39.4)	0.39 (0.17 – 0.89)	**0.023**
	No	36 (80.0)	57 (60.6)	1	
Other medications	Yes	4 (8.9)	12 (12.8)	1	
	No	41 (91.1)	82 (87.2)	1.46 (0.44 – 4.82)	0.503

*Fisher’s exact test was used where we had a cell count less than 5

- Inestimable

### Anti-epileptic drug therapy

**Fig. 2:**
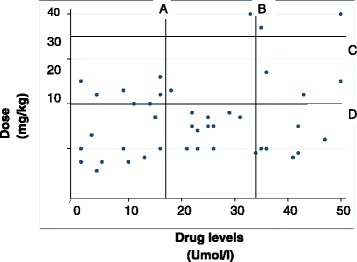
Scatter plot of Carbamazepine drug doses vs. drug levels. The majority of the study participants has sub-optimal drug levels despite taking the recommended drug doses. **a** – Minimum therapeutic dose of carbamazepine. **b** – Maximum therapeutic dose of carbamazepine. **c** – Maximum dose recommended for carbamazepine. **d** – Minimum dose recommended for carbamazepine. Recommended maximum maintenance dose for Carbamazepine is 20 – 25 mg/kg; the therapeutic range for Carbamazepine combination therapy is 16.8 – 33.8 μmol/l
  Thirteen children 13/42 (31.0 %) had drug levels within the therapeutic range. Of these; one child was on the recommended maintenance drug dose, another had a higher than recommended drug dose while 11 children were on lower than the recommended maintenance drug doses. In addition, 12 children 12/42 (28.5 %) had drug levels above the therapeutic range. Of these; 2 had higher than recommended drug doses, 3 were receiving recommended maintenance drug doses and 7 had less than the recommended drug doses. Only one child (2.4 %) was on both the recommended maintenance drug dose and had drug levels within the therapeutic range.
iii. Adherence to anti-epileptic drug therapy
  Adherence was assessed using a self-report of a three, seven and 28 day- recall. The mean adherence was 82.7 %. There was no statistically significant difference in adherence between children using monotherapy and those on multi AED. However, the majority of caretakers 93/139 (66.9 %) reported that at least on one occasion during the course of their child’s treatment, drugs were not available either in the clinic’s pharmacy or the hospital’s central pharmacy where medications are provided at no cost necessitating purchase from either drug shops or private pharmacies.
iv. Side effect profile
  Caretakers of thirty two 32/139 (23.0 %) children reported their child had ever experienced a side effect while on treatment with AEDs however at the time of the survey based on symptoms and examination, no child had clinically evident adverse events. Side effects previously experienced included; nausea/vomiting – 5, headache – 9, drowsiness – 8, hyperactivity – 2, abdominal discomfort – 3, impaired school performance – 5.

i. Doses of first anti-epileptic drug used
  On average, children using multiple AEDs were prescribed a smaller dose of their first AED compared to children on monotherapy; sodium valproate – median dose 12 mg/kg/day (IQR 10–25) vs. 18.5 mg/kg/day (IQR 10–29), carbamazepine – median dose 13 mg/kg/day (IQR 10–20) vs. 10 mg/kg/day vs. 13 mg (IQR 5.5-16) and Phenobarbitone 2 mg/kg/day (IQR 2–7.5) vs. 4 mg/kg/day (IQR 2.5-7.5). The differences in drug doses were not statistically significant between the two groups.
ii. Prescribed drug doses and serum drug levels for children on multi-therapy
  The most commonly used drug in this study population for both monotherapy and multi-therapy was carbamazepine. Regarding those on multi-therapy, forty two children 42/45 (93.3 %) were using carbamazepine in their drug combinations; 36/45 were on carbamazepine and sodium valproate, 4/45 were on carbamazepine and phenobarbitone while 2/45 were on carbamazepine, sodium valproate and phenobarbitone. Overall, the majority of children 83/139 (59.7 %) had sub-therapeutic serum drug levels including 19/45 42.2 % of those on multi-therapy. However, since it has an established dose–drug level relationship and because of its frequent use, we determined and used serum levels of carbamazepine as a representative of other drugs in analysis. There was variability in drug doses and serum drug levels in children using multi-therapy (Fig. 2). A total of only 11 children 11/42 (26.2 %) were receiving adequate maintenance drug doses. Seventeen children, 17/42 (40.5 %) had drug levels below the therapeutic range. Of these children; none had a higher than recommended drug dose, seven were receiving the recommended maintenance drug dose while 10 were receiving a dose lower than the recommended maintenance drug dose.

### Factors associated with multiple anti-epileptic drug therapy

On bivariate analysis, poor seizure control defined as one or more seizures in the previous six months (*p* < 0.001), a focal neurological deficit (*p* < 0.001), history of status epilepticus (*p* = 0.027), initiation on AED treatment with two drugs (*p* = 0.016), and treatment with sodium valproate (*p* = 0.023) were associated with multiple AED but only poor seizure control (OR^a^ 3.93 95 % CI 1.66 – 9.31, *p* = 0.002) and presence of a focal neurological deficit (OR^a^ 3.86 95 % CI 1.31 – 11.48, *p* = 0.014) were independently associated with using multiple AEDs. Children using sodium valproate (OR^a^ 0.28 95 % CI 0.11 – 0.71, *p* = 0.007) were less likely to be using multiple AED therapy.

### Seizure control in children using anti-epileptic drug therapy

**Fig. 3:**
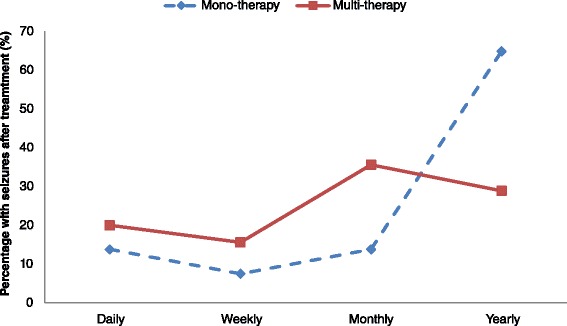
Seizure frequency on therapy. Participants on multi-therapy with AEDs were less likely to have ≤ 2 seizures in the past year compared to participants on monotherapy (*p* < 0001)

Children using multi AEDs had a higher number of daily seizures compared to children on mono therapy. Children on multi AED were also less likely to have attained good seizure control (*p* < 0.001), (Fig. 3).

## Discussion

We set out to describe multiple AED use in our clinic. The study found that 1/3 children with epilepsy in the clinic were on multiple AEDs which was also associated with poor seizure control and the presence of focal neurologic deficits. Although adherence was reported as good, the  majority of patients had sub-therapeutic serum drug levels.

The frequency of multiple AED use in this cohort is similar to that described in two previous European studies [20, 21] but markedly less than the 50 % in a Scottish study that included both children and adults [22]. It is possible that in some of our patients, the second AED could have been introduced too early since on average, children using multi-therapy in our study received a lower dose of the first AED compared to children on mono-therapy. This may have been due to prescribers not realizing the need to gradually increase the dose of the first AED before tapering off and introducing a second AED before managing children with two or more drugs. There are country guidelines for the management of epilepsy with recommended drugs and doses for each seizure type but these do not emphasize monotherapy and even then are not readily available, not even in the PNC which is in a national referral hospital. In addition, there is usually no continuity of care since senior house officers run the clinic on a rotational basis. There is evidence that optimizing the dose of monotherapy AEDs may control seemingly refractory cases of epilepsy [23]. If some of our patients were prematurely initiated on multiple AED therapy then our study could have overestimated the number of children who actually require multi AEDs to control their seizures. This has an impact on adherence, drug-drug interactions with the potential of increased adverse effects, increased costs and the probability that seizures may not be controlled even with multiple drug use [24].

## Factors associated with use of multiple AED therapy

### Clinical characteristics

Seizure onset in the majority of our patients was in the first year of life and the median duration of seizures at the time of the study was four years. Based on the clinical, neurophysiologic and imaging features, 2/3 of the patients had symptomatic or probably symptomatic epilepsy. These findings are similar to those in several cohorts in Africa and in other developing countries and have been attributed to a higher incidence of acquired brain injury from adverse perinatal events and CNS infections in resource limited settings [4, 25]. The association of multi AED use with focal neurologic signs or symptomatic or probably symptomatic epilepsy is also consistent with other studies around the world in which children with such seizures were less likely to achieve seizure control with the first attempt at monotherapy [13, 20, 26].

### Anti-epileptic drugs

We observed that children initiated on sodium valproate as the first line AED were less likely to be using multiple AED. This may be attributed to the fact that carbamazepine was the first line AED in patients with the potentially more difficult to treat symptomatic seizures (hence a higher potential of treatment failure). However, the same could have been due to the broad spectrum nature of sodium valproate. Sodium valproate has demonstrated efficacy in the treatment of an array of seizure types including; generalized idiopathic seizures, focal seizures, and epileptic syndromes like Lennox-Gastaut [27]. Two hospital based studies in the USA demonstrated that children with focal seizures and in particular, those with secondary generalization had improved seizure control after sodium valproate was introduced as monotherapy despite failing on earlier attempts with carbamazepine, phenytoin and phenobarbitone [28, 29]. Another study in the same country also demonstrated that patients with co-morbid neurological impairments could successfully be weaned off multiple AED therapy to sodium valproate monotherapy [30].

### Association between multiple drug use and seizure control

Study participants managed with multiple AEDs were almost four times more likely to have poor seizure control compared to those using monotherapy; 29 % reported good seizure control. This may have been due to the high frequency of focal seizures and the high rates of symptomatic epilepsy which may be due to underlying brain structural abnormalities. In addition, inadequate drug doses with sub-therapeutic serum drug levels might also have contributed to the poor seizure control in our patients. Proper education of health care providers regarding adequate dosing and side effect monitoring for AEDs is required to address issues of low dose polytherapy among people living with epilepsy. Advocating and engaging ministry of health to ensure that appropriate AEDs are readily available, introduction of newer broad spectrum AEDS such as Levetiracetam and the introduction of health insurance or cost sharing may help mitigate these challenges. Inadequate control of seizures increases the risk of social ostracism and stigmatization especially in low resource settings. In many African countries, epilepsy is still considered contagious and supernatural powers are often quoted as its cause. As a result, parents often seek spiritual healing before seeking medical care and may continue to do so even while their children are in care often times missing appointments and drugs, which in addition to lack of resources for transport to the hospital and the purchase of necessary drugs may adversely affect follow up and treatment outcomes [31].

### Study limitations

First, the study partly relied on participant recall to assess seizure control and self-report to assess adherence to AEDs. However, the presence of definite serum drug levels would have limited these effects. Secondly, formal assessments for hearing, vision, speech and language impairments were not carried out systematically in all patients but only in selected patients where concern had been expressed. This could have led to an under representation of patients with neurological impairment. Thirdly, treatment is provided freely at all public health facilities within the country, the prescribed drugs depend on the available class and majority of our patients lack out of pocket funds to maintain adequate dosing.

## Conclusions

One third of children with epilepsy attending the epilepsy clinics at Mulago hospital are being managed with multiple Anti-Epileptic drug therapy. However many of these children might have been inappropriately initiated onto multiple AEDs as they were on lower than recommended maintenance doses. This might be related to lack of adequate AED supply and availability in our settings. Training health workers to use appropriate treatment guidelines and recommended drugs and their doses, patient education, the use objective measures of adherence monitoring and increased access to monitoring drug levels may improve the rational use AEDs and seizure control. Strategies are also required to support the continuous availability of supplies of AEDs and to increase the range of anti-epilepsy treatment options in the country.
